# Solitary Hepatic Lymphangioma

**DOI:** 10.1155/1994/63638

**Published:** 1994

**Authors:** M. Stavropoulos, C. Vagianos, C. D. Scopa, C. Dragotis, J. Androulakis

**Affiliations:** 1 Department of Surgery University of Patras Patras Medical School Greece; 2 Department of Surgical Pathology University of Patras Patras Medical School Greece

## Abstract

Hepatic lymphangiomas are extremely rare; moreover cystic lymphangiomas usually arise in areas such as the neck and axilla, where loose connective tissue allows the expansion of lymphatic channels. The case of a 65-year old male is described, who presented with a solitary lymphangioma in the liver. The lesion was discovered incidentally and due to diagnostic uncertainty was removed surgically. A short review of histology, clinical presentation and preoperative diagnostic difficulties of hepatic lymphangiomas is given.


*HPB Surgery*, 1994, Vol. 8, pp. 33–36
Reprints available directly from the publisher
Photocopying permitted by license only

© 1994 Harwood Academic Publishers GmbH
Printed in Malaysia

### Solitary Hepatic Lymphangioma

### A Rare Benign Tumour: A Case Report

M. STAVROPOULOS*, C. VAGIANOS*, C. D. SCOPA†, C. DRAGOTIS* and J. ANDROULAKIS*

* Department of Surgery, University of Patras, Patras Medical School, Greece
† Department of Surgical Pathology, University of Patras, Patras Medical School, Greece

Hepatic lymphangiomas are extremely rare; moreover cystic lymphangiomas usually arise in areas such as the neck and axilla, where loose connective tissue allows the expansion of lymphatic channels. The case of a 65-year old male is described, who presented with a solitary lymphangioma in the liver. The lesion was discovered incidentally and due to diagnostic uncertainty was removed surgically. A short review of histology, clinical presentation and preoperative diagnostic difficulties of hepatic lymphangiomas is given.

KEY WORDS: Liver lymphangioma cystic hygroma

### INTRODUCTION

Lymphangiomas are probably congenital malformations of the lymphatic system, composed of dilated endothelial—lined spaces of varying sizes, containing lymph[1]. In 95% of the cases, lymphangiomas are located in the neck or the axilla while the remaining 5% are scattered throughout the body[2]. Hepatic lymphangiomas (HL) are very uncommon and after the first description by Ziegler in 1892[3], only a few cases have been documented[4–15]. They are usually observed in children or adolescents[16], and liver involvement, if any, is usually part of diffuse lymphangiomatosis of multiple organs, including spleen, kidneys, bones, gastrointestinal tract, mesentery, mediastinum, lungs, pleura, pericardium and soft tissues[2–4,15–18]. This report describes the case of a solitary HL in an adult, the presenting symptoms were at first attributed to chronic calculous cholecystitis.

### CASE REPORT

A 64-year old male, with a history of heterozygous-$\beta$-thalassaemia, presented, complaining of colicky epigastric and right hypohondrial pain. His upper right abdomen was slightly tender, and a soft mass was palpable in the epigastrium. Total serum bilirubin was 3.2 mg/dl, with the conjugated fraction 2.5 ml/dl. Hepatic biochemistry, $\alpha$-fetoprotein, carcinoembrionic antigen and anti-echinococcal antibodies, were normal. Hepatic ultrasound (U/S) and computerized-tomography (CT-scan) examination, demonstrated a cystic, thick-walled mass, with a solid component (Figure 1). At laparotomy, a soft, well circumscribed hepatic tumour, was discovered in segment III. The gallbladder contained multiple stones, and no other pathology was detected in the abdomen. The tumour was removed by an atypical hepatic resection. Cholecystectomy was performed and an intraoperative cholangiogram revealed a normal biliary tree. The patient was discharged on the 7th postoperative day, and two years later, he is in good health, with normal liver biochemistry, except for a slightly elevated bilirubin. On macroscopic examination, the tumour was a sharply defined, partially encapsulated solitary cyst, 4 cm in greatest diameter and located in the liver parenchyma. The cyst had a thick (8 mm), sponge-like, gray-white wall and was filled with chylous-like watery fluid, admixed with a small amount of blood. Microscopically, the wall of the cyst consisted of a meshwork of large, empty lymphatic channels, lined with flattened endothelial cells, resting on a loose myxoid connective tissue stroma (Figure 2). The liver parenchyma surrounding

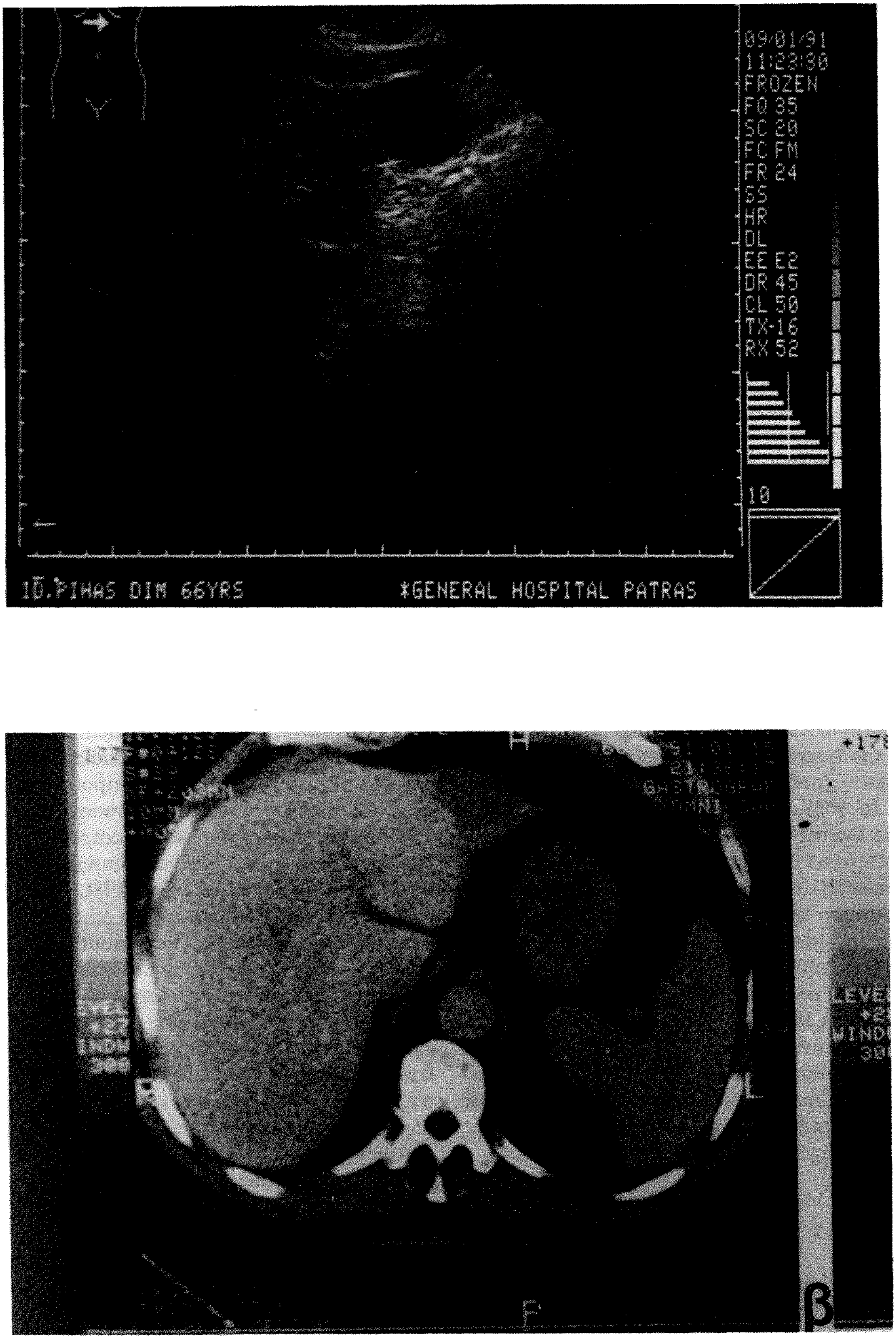

**Figure 1** Ultrasound (a) and computerized tomography (b) of the liver. They revealed the lesion in the lateral segment of the left lobe.

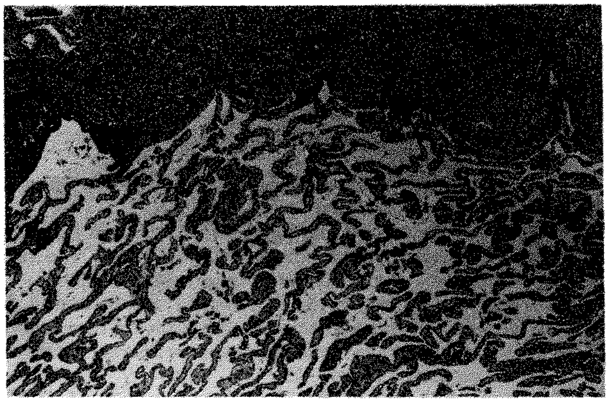

**Figure 2** Liver lymphangioma: Freely anastomosing lymphatic spaces (HE × 100).

the cyst, was compressed and hemorrhagic. The histologic diagnosis was that of a cavernous lymphangioma of the liver.

### DISCUSSION

The majority of lymphangiomas seem to represent developmental lesions, occurring relatively early in life. They are generally considered as areas of localized lymphatic stasis, due to congenital blockage of the regional lymphatic drainage[19,20]. Hepatic lymphangiomas are extremely rare tumours. Either as solitary hepatic tumours or as hepatic localization of diffuse lymphangiomatosis, no more than 42 cases have been reported in the literature[4–15]. Although there is some controversy regarding the hamartomatous, neoplastic, or lympangiectasic nature of lymphangiomas, all authors agree that they are benign tumours without malignant potential[4]. Histologically they are composed of lympatic spaces lined by attenuated endothelium and filled with proteinaceous fluid containing lymphocytes. Occasionally erythrocytes may also be present[1]. Traditionally lymphangiomas are divided, depending upon the size of the lymphatic spaces, into three groups; capillary or simple, cavernous and cystic[21]. A combination of vascular anomalies, namely lymphangiomas and hemangiomas, can coexist[22].

The morphology of the reported case of HL was atypical, with cavernous and cystic elements. Such cystic lymphangiomas are located almost exclusively in the neck where they are called "cystic hygromas"[19]. This lesion was either cystic from the beginning, or a typical cavernous lymphangioma that underwent central cystic degeneration.

The clinical presentation of HL is in general atypical[1,15], and pain, if any, is due to tension of the liver by the enlarging lesion[1,15,16]. In our case, it is uncertain whether the patient's complaints were due to the lymphangioma or to coexisting cholecystitis.

The typical U/S, CT-scan and MRI appearance of HL, is that of a cystic or multicystic hepatic mass with internal septations[23], and it is difficult to differentiate it, from necrotic hepatic metastases, or hepatic hydatidosis. It has been suggested that, these investigations are used only for evaluation of the extrahepatic extend of the disease, in cases of diffuse lymphangiomatosis[24]. In our patient, the preoperative investigations were unable to identify the nature of the hepatic mass. A diagnostic fine needle aspiration biopsy was not attempted, because of the possibility of hydatid disease and the need for an exploratory laparotomy was clear.

In conclusion, the discovery of an hepatic lesion, symptomatic or not, requires identification of the nature of the mass and a decision on further treatment[25]. However, an accurate preoperative diagnosis is extremely difficult, and no imaging technique or tumour markers, can identify the nature of the hepatic mass that uncommonly happens to be a lymphangioma. Laparoscopy and puncture biopsies, have been suggested as the only way to achieve a reliable diagnosis of HL[26]. If the diagnosis is obtained, since no malignant transformation of the tumour has been described for the asymptomatic or moderately symptomatic patients, no treatment is necessary. However, when the diagnosis is in doubt, or if the patient develops annoying symptomatology, surgical treatment by the most conservative hepatic resection is required. The prognosis following successful surgical removal is excellent.

### REFERENCES

1. Enzinger, F. M. and Weiss, S. W. (1988) Soft tissue tumours, 2nd ed, Mosby, St-Louis, pp 614–637. St-Louis: Mosby.
2. Singh, S., Baboo, M. L. and Pathak, I. C. (1971) Cystic lymphangioma in children: report of 32 cases including lesions at rare sites. *Surgery*, **69**, 947–951.
3. Ninard, B. (1959) Tumeur du fois. pp 453–55. Paris, Le Francois.
4. Barnes, P. A., Thomas, J. L. and Bernardino, M. E. (1981) Pittfalls in the diagnosis of hepatic cysts by computed tomography. *Radiology*, **141**, 129–133.
5. Durnov, L. A. and Pashkov, I. U. V. (1978) Traitement des tumeurs primitives du foîe chez l' enfant. *Khirurgiia (Mosk)*, **11**, 128–2, (en russe).
6. Jovanoviec, D. M., Opriec, M., Milanoviec, B. and Radǒjkoviec, P. (1980) Clistiucni limfangiom jetre. *Acta Chirurgica Jugoslava*, **27**, 71–76.
7. Moran Penco, J. M., Borella, F., Garibaldi, R., Toma, P. and Dodero, P. (1983) Hemangiolinfangioma hepatico en el lactante. *Annals Esp. Pediatr.*, **19**, 411–413.

8. Prikhodchenko, V. V. (1985) Lymphangiomes hepatiques de l' enfant. *Vopr. Okhr. Materin. Det.*, **30**, 38–41, (en russe).
9. Rumpe, P. and Mannfeld, V. (1972) Zystisches Lymphangiom der Leber beim Kind. *Zentralbl. Chir.*, **29**, 1060–1063.
10. Sathyavagiswaran, L. and Sherwin, R. P. (1989) Acute and chronic pericholangiolitis in association with multifocal hepatic lymphangiomatosis. *Human Pathology*, **20**, 601–603.
11. Spachi, A., Mazzone, A., Pezzali, M., Ricevuti, G., Luliri, P. and Rizzo, S. G. (1986) Liver lymphangiomaa rare benign tumour. A case presentation. *Med. Biol. Environ.*, **14**, 441–445.
12. Tahon, O. (1988) Lymphangiome du foie. A propos de deux cas et revue de la literature. These Medecine, Amiens.
13. Talegon Melendez, A., Guttierez-Alviz, J. M., Olloqui Martin, E., Piero de las Heras, J., Peres Vega, H. and Mauduit Astolfi, I. (1987) Linfangioma hepeatico neonatale. *Radiologia*, **29**, 523–526.
14. Tramonti, M., Parisi, L., Del Moro, J. and Dal Pozzo, G. (1982) Un caso di linfangioma cistico del fegato con ultrasonografia e tomodensitometria. *Radiologia Medica* (Torino), **68**, 667–668.
15. Delamarre, J., Lamblin, G., Sevestre, H., Deschepper, B., Jouet-Gondry, C., Davion, T. and Capron, J. P. (1990) Lymphangiome caverneux du foie. Etude de 2 cas et revue de la litterature. *Gastroenterologie Clinique et Biologique*, **14**, 576–580.
16. Asch, M. J., Cohen, A. H. and Moore, T. C. (1974) Hepatic and splenic lymphangiomatosis with skeletal involvement: report of a case and review of the literature. *Surgery*, **76**, 334–339.
17. Schuster, S. R. and Gang, D. R. (1980) Case records of the Massachusetts General Hospital. An 11-year-old girl with multiple osteolytic lesions and a mediastinal mass. *New England Journal of Medicine*, **303**, 270–276.
18. Larson, D. L., Myhre, B. A. and Schmidl, E. R. (1961) Lymphangioma in unusual sites: spleen, mesentery, retroperitoneum, me diastinum, and the greater omentum. *Wisconsin Medical Journal*, **60**, 279–287.
19. Bill, A. H. and Sumner, D. S. (1965) A unified concept of lymphangioma and cystic hygroma. *Surgery Gynecology and Obstetrics*, **120**, 79–86.
20 Goart, S. (1966) Embryological significance of lymphangiomas. *Archives of Diseases in Childhood*, **41**, 204–206.
21. Asch, M. J., Cohen, A. H. and Moore, T. C. (1974) Hepatic and splenic lymphangiomatosis with skeletal involvement: report of a case and review of the literature. *Surgery*, **76**, 335–339.
22. Bardeguez, A., Chatterjee, M., Topdino, M. and Sicuranza, B. (1990) Systemic cystic angiomatosis in pregnancy; A case presentation and review of the literature. *American Journal of Obstetrics and Gynecology*, **163**, 42–45.
23. Blumhagen, J. D., Wood. B. J. and Rosenbaum, D. M. (1987) Sonographic evaluation of abdominaly lymphangiomas in children. *Journal of Ultrasound Medicine* **6**, 487–495.
24. Cutillo, D. P., Swatbe, K. C., Cucco, J. and Dougan, H. (1989) Case Report. CT and MR imaging in cystic abdominal lymphangiomatosis. *Journal of Computed Assistant Tomography*, **13**, 534–536.
25. Little, J. M., Kenny, J. and Hollands, M. J. (1990) Hepatic incidentaloma cidealoma: A modern problem. *World Journal of Surgery*, **14**, 448–451.
26. Steenbergen, W. V., Joosten, E., Marchal, G., Baert, A., Vanstapel, M. J., Desmet, V., Wijnants, P. and De Groote, J. (1985) Hepatic lympangiomatosis. Report of a case and review of the literature. *Gastroenterology*, **88**, 1968–1972.

